# COVID-19 test-to-stay program for K-12 schools: Opt-in versus opt-out consent model

**DOI:** 10.1016/j.isci.2023.108770

**Published:** 2023-12-20

**Authors:** Anton Ivanov, Ujjal Kumar Mukherjee, Subhonmesh Bose, Sridhar Seshadri, Ronald Watkins, Albert Charles England, Jacqueline Suriano, Mehmet Eren Ahsen, Sebastian Souyris

**Affiliations:** 1 Department of Business Administration, University of Illinois at Urbana-Champaign, Champaign, IL 61821, USA; 2 Electrical and Computer Engineering, University of Illinois at Urbana-Champaign, Champaign, IL 61821, USA; 3 Shield Illinois, University of Illinois System, Champaign, IL 61821, USA; 4 OSF HealthCare Heart of Mary Medical Center, Urbana, IL 61801, USA; 5 Lally School of Management, Rensselaer Polytechnic Institute, Troy, NY 12180, USA; 6 Health Innovation Professor Carle Illinois College of Medicine, University of Illinois at Urbana-Champaign, USA

**Keywords:** System management, Social sciences, Research methodology social sciences

## Abstract

The Centers for Disease Control and Prevention promoted the Test-to-Stay (TTS) program to facilitate in-person instruction in K-12 schools during COVID-19. This program delineates guidelines for schools to regularly test students and staff to minimize risks of infection transmission. TTS enrollment can be implemented via two different consent models: opt-in, in which students do not test regularly by default, and the opposite, opt-out model. We study the impacts of the two enrollment approaches on testing and positivity rates with data from 259 schools in Illinois. Our results indicate that after controlling for other covariates, schools following the opt-out model are associated with 84% higher testing rate and 30% lower positivity rate. If all schools adopted the opt-out model, 20% of the total lost school days could have been saved. The lower positivity rate among the opt-out group is largely explained by the higher testing rate in these schools, a manifestation of status quo bias.

## Highlights

• Schools adopting opt-out model experience an 84% higher testing rate, on average
• Generally, schools adopting opt-out model experience a 30% lower positivity rate
• Lower positivity rate among the opt-out group is explained by the higher testing rate
• If all schools adopted opt-out model 20% of total lost school days could have been saved

## Graphical abstract

**Figure:**
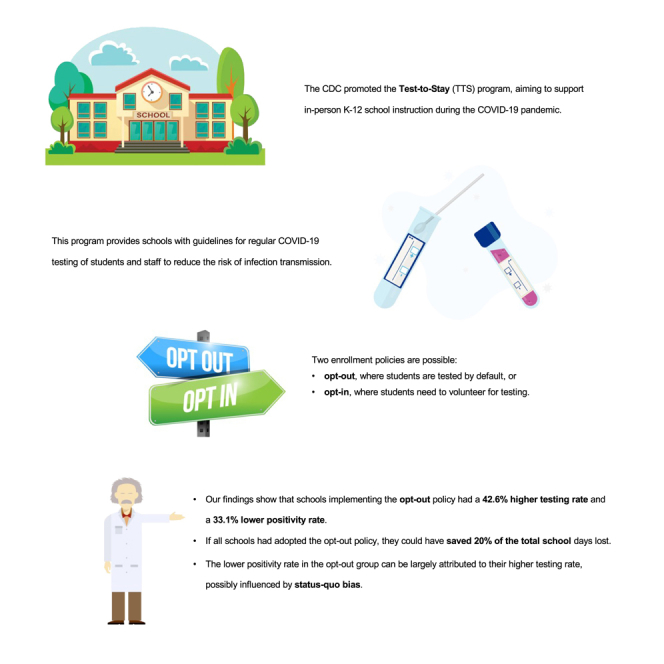

## Introduction

Many educational institutions moved to online classes during the COVID-19 pandemic to minimize the potential risks of infection from in-person interactions. Prolonged distance learning, however, poses serious challenges.^1^^,^^2^^,^^3^ Teachers have consistently complained about low student engagement in online classrooms. Moreover, the online modality differently affects students from different socio-economic backgrounds.^4^^,^^5^ Regular testing is a critical component in ensuring the safety and continuity of in-person education.^6^ The Centers for Disease Control and Prevention proposed the Test-to-Stay (TTS) program, a COVID-19 testing program for educational institutions to enable safe resumption of in-person classes. Per the TTS program, schools that choose to participate have the option to perform contact tracing and testing on individuals who are willing, enabling them to remain in in-person classes without the need for quarantine. The Illinois Department of Public Health expanded free COVID-19 testing to all K-12 public schools in Illinois willing to participate in the TTS program. Participating schools could utilize the SHIELD Illinois saliva-based test developed by the University of Illinois Urbana-Champaign (UIUC), which is able to detect severe acute respiratory syndrome coronavirus 2 (SARS-CoV-2) and its variants in symptomatic, pre-symptomatic, and asymptomatic individuals. In Illinois, the school district authority, which may encompass multiple schools, is responsible for making the decisions on adoption and implementation of TTS. The school districts could choose to implement the TTS program using either of the two alternate consent models, namely, the *opt-in* and *opt-out models*.^7^^,^^8^ Under the opt-in model, students were by default not tested unless the students (or their legal guardians) explicitly chose to enroll to be tested regularly regardless of exhibited symptoms or after being traced as a close contact of a detected individual. Under the opt-out model, in contrast, the students are automatically enrolled in regular testing unless they (or their legal guardian) explicitly choose not to enroll. The sequence of events in the TTS program is as follows: *(i)* school districts choose to adopt TTS and choose the consent model, *(ii)* students (or their legal guardians) are given the option to formally consent to enroll in testing for schools following the opt-in model, or the option to formally withdraw consent from enrolling in testing for schools following the opt-out model, *(iii)* enrolled students are regularly tested at a pre-determined frequency, *(iv)* infected individuals detected in the testing process are required to quarantine for a pre-determined period (15 days), *(v)* the close contacts of the detected individuals are traced, (vii) contacts who are not enrolled in testing are required to quarantine, and, *(vi)* finally, contacts who are enrolled in testing under the TTS program are tested, and they continue to attend in-person classes as long as they test negative, else, they quarantine.

Debates have ensued^9^^,^^10^^,^^11^ to evaluate the pros and cons of the consent models for enrolling students in the TTS program. Teacher unions were concerned with health and safety due to potentially low rates of testing in providing the choice not to test, which equally exists in both consent models.^12^ In contrast, student and parent groups were concerned with exercising freedom regarding testing and the privacy of student testing data due to potential contact tracing.^13^ Notwithstanding privacy concerns,^14^ policy-level debates focus on the question *which of the two consent models for enrollment is more effective in mitigating the spread of the pandemic?* Just as forecasting the likelihood and impact of disruptions is a crucial element of the effective management of supply chains,^15^ the ability to evaluate the effectiveness of pandemic mitigation measures such as the TTS program is a key aspect in ensuring the safe functioning of educational institutions such as K-12 schools. In this paper, we use data from the K-12 TTS program in the state of Illinois, USA, to evaluate the relative effectiveness of the two enrollment policies, namely, the opt-in and opt-out models.

To evaluate the efficacy of the two consent models, we compute two different performance measures, namely, *(i)* the *testing rate*, the ratio of the total number of tests conducted in a week at a school to the school size (including both student population and staff), and *(ii)* the *test positivity rate*, the ratio of the total number of COVID-19 positive test results in a week at a school to the total number of tests conducted in that school in the week. Test positivity is considered a strong correlate of school infectivity.^16^ Our regression analysis results demonstrate that at the K-12 schools in Illinois, the opt-out consent model is associated with an 84% higher testing rate and a 30% lower test positivity rate than the opt-in model. These estimates translate to a 1.84 times higher testing rate and 0.69 times lower positivity rate at schools in the opt-out group compared to schools in the opt-in group. The aggregate-level relationship is supported across multiple school types (elementary, middle, high) and across metropolitan and other locations. Additionally, our analysis demonstrates that the reduced positivity rates observed in schools with an opt-out model can be predominantly attributed to their notably higher testing rates. We posit that status quo bias is a cognitive factor that may be responsible for the different testing rates between the two consent models. Status quo bias reflects the inclination to choose the default option from a range of possible options.^17^ The study provides valuable insights for policymakers, including schools, school districts, and state-level administrators, urging them to develop strategies that promote the adoption of practices aligned with anticipated public behavior. To our knowledge, this is one of the first studies using field data across a large number of K-12 schools to assess TTS policy implementation from the alternative consent model for enrollment in TTS programs.

## Results

### Effect of consent model

Table 1 shows the descriptive statistics of the data sample. According to the results, the testing rate averaged over all schools and all weeks for the opt-out group of schools is 46.78%, while the corresponding average testing rate for the opt-in group is 31.34%. These estimates translate to a 15.44% higher overall testing rate among schools in the opt-out group compared to the schools in the opt-in group. The average positivity rate is 1.81% for the schools in the opt-out group and 2.40% for the schools in the opt-in group. These numbers indicate that the opt-out group had a 0.59% lower positivity rate than the opt-in group. These estimates translate to a 1.49 times higher testing rate and 0.75 times lower positivity rate at schools in the opt-out group compared to those in the opt-in group.

**Table 1:** Descriptive statistics of the data from the SHIELD Program (SE in parentheses)

Group	Sample	Test rate (%)	Positivity (%)	School size	Community vaccination (%)	Community positivity (%)
	259	38.75 (28.02)	2.12 (3.79)	708.22 (532.45)	70.71 (26.99)	0.75 (0.44)
***Enrollment policy***						
Opt-in	144	31.34 (26.29)	2.40 (3.59)	647.03 (518.22)	68.67 (25.74)	0.82 (0.46)
Opt-out	115	46.78 (27.65)	1.81 (3.97)	774.48 (540.43)	72.87 (28.13)	0.68 (0.390)
***Location***						
Metropolitan	221	39.80 (28.13)	1.88 (3.11)	735.62 (557.52)	72.99 (27.34)	0.70 (0.035)
Non-metropolitan	38	32.80 (26.72)	3.51 (6.24)	551.93 (316.39)	57.84 (20.71)	1.05 (0.67)
***School Type***						
Elementary	165	46.18 (26.26)	1.63 (2.80)	475.30 (176.95)	72.22 (27.01)	0.70 (0.36)
Middle	42	35.06 (25.12)	1.76 (2.17)	714.88 (246.97)	72.73 (28.12)	0.73 (0.36)
High	52	20.95 (26.95)	3.86 (6.21)	1370.34 (792.42)	64.23 (24.93)	0.93 (0.63)

**Figure 1:**
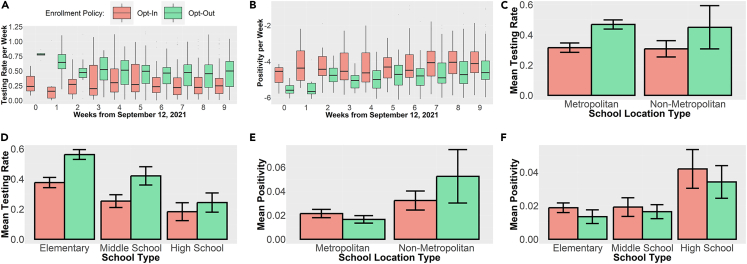
Plot of weekly outcomes and aggregate outcomes by enrollment policy (A) Weekly testing rate by enrollment policy. (B) Weekly positivity rate by enrollment policy. (C) Aggregate testing rate by school location type. (D) Aggregate testing rate by school type. (E) Aggregated positivity rate by school location type. (F) Aggregate positivity rate by school type. Error bars represent 95% confidence interval.

Furthermore, Figures 1A and 1B highlight a consistent difference between opt-in and opt-out enrollment approaches within the weekly data. Overall, Figure 1A indicates that the opt-out policy leads to higher weekly testing rates than does the opt-in policy. Similarly, Figure 1B indicates that the weekly test positivity for the opt-out group is consistently lower than that of the opt-in group across all weeks. Table 2 provides the statistics pertaining to the test of difference between the mean/median testing rates and test positivity rates between schools in the two groups. Evidently, the differences are statistically significant at p < 0.05 for almost all weeks with a few exceptions. These results indicate that the opt-out enrollment policy is consistently associated with higher testing rates and lower positivity rates.

**Table 2:** Weekly testing and positivity rates (*N* = 259)

	Mean	Mean	Mean	Median	Median	Median
Week	Opt-in	Opt-out	Difference	Opt-in	Opt-out	Difference
*Testing*						
0	0.30	0.78	0.48#	0.24	0.77	0.54∗
1	0.26	0.65	0.39#	0.16	0.64	0.49∗
2	0.28	0.49	0.22∗	0.27	0.47	0.20∗∗
3	0.34	0.50	0.17∗	0.20	0.52	0.32∗
4	0.36	0.47	0.11	0.30	0.51	0.21#
5	0.35	0.49	0.13#	0.27	0.49	0.23∗
6	0.29	0.45	0.15∗∗	0.23	0.46	0.23∗∗∗
7	0.32	0.45	0.12∗	0.22	0.49	0.28∗
8	0.29	0.43	0.14∗∗	0.22	0.47	0.24∗∗
9	0.30	0.46	0.15∗∗	0.25	0.50	0.25∗∗
***Positivity***						
0	−5.01	−5.87	−0.86	0.01	0.00	−0.01
1	−4.24	−5.75	−1.51∗	0.01	0.00	−0.01∗
2	−4.40	−4.92	−0.52#	0.01	0.01	−0.00
3	−4.41	−5.23	−0.82∗∗	0.01	0.01	−0.01∗∗
4	−4.48	−5.13	−0.66∗∗	0.01	0.01	−0.00∗∗
5	−4.43	−4.84	−0.41#	0.01	0.01	−0.01∗
6	−4.25	−4.97	−0.72∗∗	0.01	0.01	−0.00∗∗
7	−4.18	−4.72	−0.54∗	0.02	0.01	−0.01∗∗
8	−4.15	−4.62	−0.48∗	0.02	0.01	−0.01∗∗
9	−4.12	−4.47	−0.34∗	0.02	0.01	−0.01∗∗

Note: *∗∗∗*p *< 0.001, ∗∗*p *< 0.01, ∗*p *< 0.05, #*p *< 0.1*.

Differences of mean and median are computed as (opt-out – opt-in) using t test and Wilcoxon rank-sum, respectively.

Figures 1C–1F present the bar plots of the weekly testing and positivity rates across schools categorized by their location, school type, and enrollment policies. Figure 1C indicates that the opt-out policy is associated with higher testing rates than the opt-in policy for schools located in both metropolitan and non-metropolitan locations. However, Figure 1E indicates that a higher testing rate at opt-out schools results in a lower positivity rate only for metropolitan schools. Interestingly, for non-metropolitan schools, the positivity rate is higher for the opt-out group even though the testing rates are higher. One potential reason for this result is that the general community risks of infections are relatively lower in non-metropolitan areas. Testing is more important when the risk of infection is relatively higher. Focusing on school types, Figure 1D suggests that the testing rate in the opt-out group is consistently higher for elementary, middle, and high schools. Furthermore, Figure 1F suggests that the higher testing rate in the opt-out group translates to lower positivity rates across all school types. These results generally support the finding that the opt-out consent model is associated with higher testing rates and lower test positivity rates at schools.

### Controlling for the heterogeneous effects of covariates

The model-free results presented earlier do not account for other relevant factors that are likely to be associated with the outcomes. For example, it is reasonable to anticipate that positivity rates within schools might mirror the community positivity rates in the areas in which the schools are situated. To that end, we perform a regression analysis that includes the fixed effects of weeks, school types, school location, community positivity, community vaccination rates, and enrollment policy. Additionally, the regression model also includes a random effect of each school to partially account for school-level unobserved latent factors that may not have been captured in the analysis sample.

Table 3 presents the results of the regression analysis. Specifically, we consider two outcomes of interest and fit two models for each dependent variable. Model 1 (A and B) includes all variables but the content policy and, therefore, acts as the baseline model. Additionally, Model 2 includes the consent model variable. This two-step regression allows us to estimate the additional effect of the consent model. Accordingly, we conduct an analysis of variance (ANOVA) that compares the two regression models, which allows us to study the additional variation in the response variables (testing or positivity rates) explained by the consent model, after accounting for all other covariates. The variance inflation factors for all variables in all models are below 5, indicating acceptable levels of multi-collinearity. The parameter estimate for the opt-out model in Model 2A for the testing rate is 0.61 (p *value < 0.001*), suggesting that the opt-out consent model is statistically significantly associated with a higher testing rate. The consent model explains a significant additional variation in the testing rate, based on ANOVA of the regression model, with an associated χ2 statistic of 21.13 (p *value < 0.001*). For positivity rate estimates from Model 2B, the parameter estimate for the opt-out consent model is −0.36 (p *value < 0.001*), indicating that it is statistically significantly associated with lower positivity. The addition of the consent model also explains a significant additional variation with an associated χ2 statistic of 21.29 (p *value < 0.001*). In summary, regression analysis reinforces our previous conclusions regarding the impact of TTS enrollment policy on testing and positivity rates.

**Table 3:** Regression analysis results (*N* = 259)

Model	1A	1A	1A	2A	2A	2A	1B	1B	1B	2B	2B	2B
*Random effects*	*Var.*	*S. Dev.*	*Sig.*	*Var.*	*S. Dev.*	*Sig.*	*Var.*	*S. Dev.*	*Sig.*	*Var.*	*S. Dev.*	*Sig.*
School ID	1.01	1.04	∗∗∗	0.99	0.99	∗∗∗	0.63	0.79	∗∗∗	0.57	0.75	∗∗∗
Residual	0.10	0.31		0.10	0.31		0.31	0.55		0.31	0.55	
***Fixed effects***	***Est.***	***S. Err.***	***Sig.***	***Est.***	***S. Err.***	***Sig.***	***Est.***	***S. Err.***	***Sig.***	***Est.***	***S. Err.***	***Sig.***
Intercept	−1.20	0.15	∗∗∗	−0.87	0.16	∗∗∗	−4.92	0.22	∗∗∗	−4.68	0.23	∗∗∗
Week 3209	−0.03	0.15		−0.03	0.15		−0.05	0.26		−0.05	0.26	
Week 3210	−0.04	0.13		−0.04	0.13		0.24	0.23		0.23	0.23	
Week 3211	−0.12	0.13		−0.12	0.13		0.24	0.23		0.22	0.23	
Week 3212	−0.04	0.13		−0.04	0.13		0.30	0.22		0.28	0.22	
Week 3213	−0.04	0.13		−0.04	0.13		0.34	0.22		0.32	0.22	
Week 3214	−0.05	0.13		−0.05	0.13		0.27	0.22		0.27	0.22	
Week 3215	−0.07	0.13		−0.07	0.13		0.40	0.22	+	0.39	0.20	+
Week 3216	0.22	0.20		0.15	0.20		0.89	0.29	∗∗	0.98	0.29	∗∗∗
Week 3217	−0.01	0.13		−0.01	0.13		0.33	0.22		0.33	0.22	
School type (high)	−1.70	0.17	∗∗∗	−1.70	0.17	∗∗∗	0.59	0.15	∗∗∗	0.59	0.14	∗∗∗
School type (middle)	−0.34	0.19	+	−0.37	0.18	∗	0.03	0.15		0.07	0.15	
Location (metropolitan)	0.15	0.20		0.31	0.19		−0.02	0.17		−0.17	0.17	
Community positivity	0.04	0.03		0.04	0.03		0.24	0.05	∗∗∗	−0.51	0.11	∗∗∗
Community vaccination	−0.10	0.07		−0.07	0.07		−0.32	0.09	∗∗∗	0.23	0.05	∗∗∗
Consent model (opt-out)				0.61	0.13	∗∗∗				−0.36	0.08	∗∗∗
***ANOVA***	***AIC***	***Dev.***	***Chi-sq***	***DF***	***p***	***Sig.***	***AIC***	***Dev.***	***Chi-sq***	***DF***	***p***	***Sig.***
Model 1	1195.40	1161.40					1611.60	1577.61				
Model 2	1176.32	1140.30	21.13	1	0	∗∗∗	1592.31	1556.30	21.29	1	0	∗∗∗

Models: Model 1 includes all features but consent model, and Model 2 includes all features and consent model. A and B notations refer to testing rate and positivity rate outcomes, respectively. Significance code: 0 < ‘∗∗∗’ ≤ 0.001 < ‘∗∗’ ≤ 0.01 < ‘∗’ ≤ 0.05 < ‘+’ ≤ 0.1.

### Heterogeneous effects

**Figure 2:**
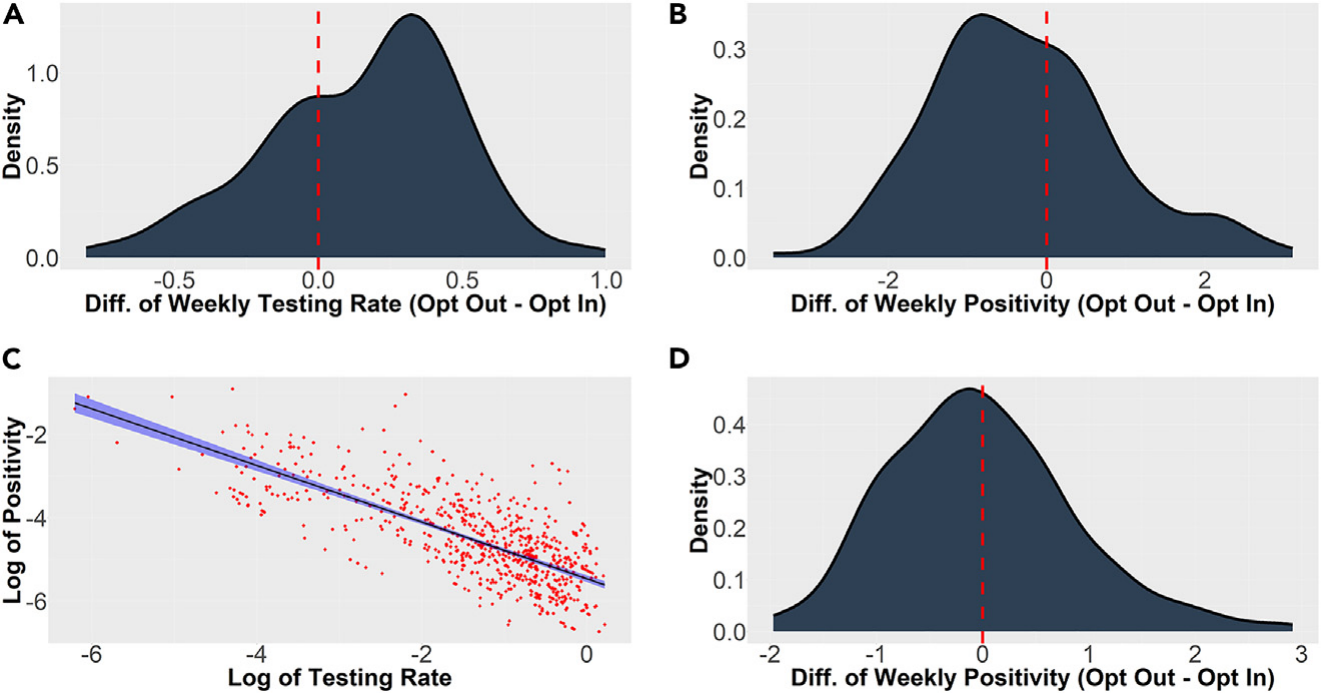
The plot of the relationship between testing rates and positivity values (A) Density of the difference between testing rates between matched samples. (B) Density of difference between positivity rates between matched samples. (C) Weekly testing rates against positivity rates across all schools and weeks. (D) Density of the difference between positivity rates between matched samples with testing rate as a covariate.

A shortcoming of the regression estimates presented earlier is that a simple regression cannot fully account for systematic baseline differences in covariates across schools in the opt-out and opt-in groups. It is likely that there exists a systematic variation in the choice of enrollment policy across school types, school locations, or schools located in locations with high versus low community positivity or vaccination rates. To establish the potential causal effect of enrollment policy on testing and positivity rates, we use a matched sample analysis. Specifically, we use coarsened exact matching (CEM) using the categorical covariates week, school type, and school location, and the continuous variables community positivity and community vaccination.^18^ In CEM, the individual observations in the opt-out (treatment) group are matched with one or more observations in the opt-in (control) group such that the categorical variables are exactly matched across the matched sample of the two groups, and the continuous variables are matched on the corresponding deciles of the distribution of the individual continuous variables.^18^ Essentially, it compares samples that adopted different consent models but are similar in all other relevant covariates. The objective of the matched sample analysis is to compute the difference in the outcome values of the observations in the opt-out (treatment) group from the corresponding matched sampled from the opt-in (control) group. We plot the distribution of the difference estimates and compute the sample average treatment effect on the treated (SATT) measured as the average difference of the outcomes of all samples in the treatment group, i.e., the opt-out group, from the corresponding matched samples in the control group, i.e., the opt-in group. Figures 2A and 2B visually represent the distribution of the individual differences in weekly testing and positivity rates. In Figure 2A, the distribution of the difference in testing rates is positively skewed, suggesting that the opt-out consent model generally yields higher testing rates than the opt-in model. In Figure 2B, the distribution of the difference in positivity of schools in the opt-out group from those of the corresponding matched schools in the opt-in group is negatively skewed, indicating lower test positivity associated with the opt-out policy. The SATT estimate for the testing rate difference corresponding to Figure 2A is 0.15 (*t-stat: 6.45,* p *value < 0.001*). Similarly, the SATT estimate for the positivity difference corresponding to Figure 2B is −0.031 (*t-stat*: *−3.81,* p *value < 0.001*). The binomial parameter estimate for the sign test of SATT of the testing rate is 0.7387 and that for the positivity rate is 0.7236. This analysis suggests a 73.87% (72.36%) probability that a randomly chosen school from the opt-out group is expected to have a higher testing rate (lower positivity rate) than another randomly selected school from the opt-in group. The analysis of the matched samples reinforces the conclusion that schools adopting the opt-out consent model generally exhibit higher testing rates and lower positivity rates than schools adopting the opt-in model.

### Endogeneity-corrected regression models

A general shortcoming of the matched sample analysis is that it can account for systematic variation in baseline only on observed covariates. However, to address potential issues related to unobserved self-selection of enrollment policy due to general variation in population sentiment toward one of the policy options, or due to unobserved variables such as masking rates and social-distancing prevalence, we adopt a model-based endogeneity-corrected regression estimate using an instrumental variable approach. To address these concerns, we begin by checking if the baseline state is similar between opt-in and opt-out groups with respect to community level health outcomes. Specifically, we conduct a t test to examine whether differences exist in community positivity and vaccination rates between the two consent models. The average community positivity rate of locations of schools in the opt-in group is 0.0071 and that of the opt-out group is 0.0062 with a t-statistic of difference in means of 5.25 (p *value* < 0.001). Similarly, the average vaccination rate of locations of schools in the opt-in group is 67.9% and that of the opt-out group is 70.3% with a t-statistic of difference in means of 1.96 (p *value* < 0.05). Interestingly, schools in locations where vaccination rates are relatively lower and community positivity rates are relatively higher choose the opt-in policy, which is associated with relatively lower testing rates and higher positivity risk at schools and vice-versa. This indicates a systematic variation in choice of enrollment policy across schools and locations based on the general sentiment toward adoption of COVID-19 preventative measures such as vaccination. Specifically, we employ a Heckman selection model, a conventional statistical technique that can be used to correct bias from non-random selection of enrollment policy. The first step of this procedure includes finding an instrumental variable (to address exclusion restriction) and other covariates to predict, using a probit model, the likelihood of selecting a given enrollment policy. To this end, building on prior literature, we identify political affiliation (measured as the percent of democratic votes cast) as a potential instrument.^19^ This instrument is theoretically sound for our case, as it shows no direct correlation with COVID-19 positivity. Although political beliefs are not in and of themselves linked with health-related outcomes, political beliefs can influence both observed and unobserved behaviors (e.g., mask-wearing, social distancing, vaccinations, etc.) that are known to affect positivity. Consequently, any association between positivity and political affiliation is likely channeled through the testing rate and consent model in our context. Upon empirical examination to validate the instrument, we observe a statistically significant non-zero direct effect of political affiliation on the selection of the consent model, while controlling for community positivity and vaccination rates. These findings are bolstered by the outcomes of the Wu-Hausman (*F* = 3.35, p *value* > 0.05) and Wu-Durbin-Hausman (*χ2* = 3.41, p *value* > 0.05) tests, which affirm the instrument’s validity. In the subsequent stage of the Heckman correction procedure, we compute the predicted inverse Mills ratio (IMR), which, in our particular case, is statistically significant (p *value* < 0.1). This observation corroborates the presence of selection bias. Finally, to correct for selection bias in estimating the effect of consent model on testing rates, we add the IMR to the second-stage model as a covariate (excluding the instrument but including other relevant covariates). Consequently, the impact of the consent model remains statistically significant (p *value* < 0.001) and in the expected direction, affirming the reliability of our conclusions (Table 4). Additionally, after incorporating this adjustment, the coefficients in the model remain largely consistent. This suggests that, if there is any endogeneity, its impact is minimal. Finally, we use a two-stage least squares (2SLS) approach with control function to address endogeneity in estimating the effect of consent model on positivity. To this end, we use political affiliation as an instrumental variable at the first stage and then capture the corrected effect of the consent model at the second stage. The effect on positivity is statistically significant (p *value* < 0.001) and aligns with our expected direction of relationship between consent model and positivity. In summary, all the different model-free and model-based estimates point toward the conclusion that the opt-out consent model is significantly associated with higher rates of testing and lower test positivity at K-12 schools.

**Table 4:** Endogeneity correction models

Outcome	Positivity rate	Testing rate
*Model*	*2SLS, RE, CF*	*Heckman*
Consent model (opt-out)	−1.16∗ (0.52)	0.58∗∗∗ (0.13)
Inverse Mills ratio	–	−1.48∗∗
*Controls*	*Yes*	*Yes*
*Week dummies*	*Yes*	*Yes*
*Wald test*	p *> 0.05*	*-*
*R-squared*	*0.15*	*0.29*

#p < 0.1, ∗p < 0.05, ∗∗p < 0.01, ∗∗∗p < 0.001.

2SLS, two-stage least squares; RE, random effects; CF, control function; Heckman, two-step Heckman selection model.

Wald test of endogeneity: H0: Treatment and outcome unobservables are uncorrelated.

### Explaining lower positivity rates via higher testing rates

We posit that the causal mechanism that connects the consent model with school positivity is the relative difference in the testing rates between the two models. Accordingly, we now demonstrate that the higher testing rates among opt-out schools largely explain the lower positivity rates. Figure 2C depicts the weekly testing rates against the weekly positivity rates at all schools and all weeks on a log-log plot. This graph yields a strong negative correlation of −0.74 between these rates (in log scale). In Figure 2D, we repeat the matched sample analysis (cf. Figures 2A and 2B) for positivity rates, but with the testing rate included among the list of matching features. Inclusion of the testing rate considerably diminishes the negative skewness of the distribution. The SATT estimate with the inclusion of testing rate as a matching variable is now reduced to −0.002 (t-stat: −0.35, p = 0.72) from an SATT estimate of −0.031 (t-stat: −3.81, p < 0.001) when the testing rate was not included as one of the matching variables. Correspondingly, the binomial sign test p value increases to 0.395, compared to less than 0.001 without testing rate as a matching feature. This suggests that consent model has a small effect on positivity rates, after accounting for the variation in the resulting testing rates from said consent models.

Finally, we conduct a Bayesian mediation analysis to formally establish the causal pathway from the consent model to school positivity rates through the testing rates. The mediation analysis embodies a model-based estimation of the conditional independence of enrollment approach and test positivity given the testing rates. Of interest to us are the average causal mediation effect (ACME) and the average direct effect (ADE). ACME computes the variation in positivity per school per week explained by consent model choice, entirely due to the mediation of the testing rate. ADE computes the variation in the positivity rate that is directly explained by the consent model, without accounting for the effect of the consent model on the testing rate. The ACME is estimated to be 0.3540 with a 95% confidence interval of (0.2563, 0.4600), corresponding to a χ2 p *value* < 0.001. The ADE is 0.0799 with a 95% confidence interval of (−0.0199, 0.1701) and has a χ2 p *value* of 0.14. Therefore, the mediation effect explains 81.6% of the effect of the enrollment approach on the variation in positivity rates. Per counterfactual analysis using Bayesian mediation, we conclude that the testing rate for the entire sample of 259 schools would have increased by 30.44%, resulting in a decrease in positivity rates by 24.93% from the observed average values, if all schools adopted the opt-out consent model. The significance of the difference in testing rate is that if all schools were to adopt the opt-out model, an additional 3,815 school days (one school day equals one student missing one day of school, considering seven days of quarantine) could have been saved over a period of 10 weeks. This represents a 20% reduction in lost school days. The extra days are preserved by preventing subsequent infections through the higher testing rate in the opt-out model.

## Discussion

Our results indicate that the opt-out consent model leads to a higher testing rate, which in turn is largely responsible for a lower positivity rate in schools, compared to the opt-in model. We believe that the higher testing rates at schools with an opt-out model are a result of status quo bias.^17^ When presented with a collection of choices, this cognitive bias captures people’s propensity to choose the default option. The roots of status quo bias are associated with the limited attention of individuals^20^ and the psychological costs associated with the efforts required to deviate from the default option.^21^ The default option in the opt-in model is not to get regularly tested, while the default option in the opt-out model is the opposite. The default option is exercised more often than the alternative, as the alternative poses an additional cognitive burden. Status quo bias has been employed to explain behavior in various contexts, including healthcare.^22^^,^^23^ Our work adds to that list.

The COVID-19 pandemic experience has underscored the importance of accounting for people’s behavioral patterns in designing interventions.^24^ Policy interventions through mandates have been widely debated and their efficacy challenged.^25^ Mask mandates have been at the epicenter of such debates. While masks themselves provide effective protection, mask mandates have witnessed mixed success due to a combination of factors.^26^ Our analysis, however, tells the opposite story for testing. Multiple mass-testing infrastructures such as the SHIELD program have been field-tested to date (per communication with the SHIELD program administrators, the cost per test has declined to $15–$30, and the testing accuracy is north of 90%), all of which demonstrate that regular testing reduced disease transmission and enabled in-person interactions, as reported in past research.^6^^,^^27^ Compared to mask-wearing, which requires continuous adherence, testing causes periodic (once or twice a week) inconvenience. Perhaps, as testing is less onerous than mask-wearing, testing mandates have proven more effective (at least within institutional settings) than mask mandates.^6^

Testing is a targeted mechanism to prevent asymptomatic transmission. The higher the testing rate, the lower the chance of infection transmission within the school. This is evidenced by the success of the TTS policy relying on the opt-out consent model for enrollment, which is largely due to testing rates that are higher than in the opt-in model. Policymakers can adopt the opt-out model to attain higher testing volumes at schools. By making regular testing the default option, this enrollment approach tests students who might not otherwise get tested due to inattention. Despite success stories, testing mandates have met with resistance. Hesitancy to subscribe to a testing mandate can stem from a myriad of concerns, such as those regarding privacy of patient data, the potential violation of personal autonomy, and questions about its efficacy.^28^ We found that schools in locations with lower vaccination rates have substantially lower chances of adopting the opt-out consent model in Illinois. Thus, a community’s beliefs and perceptions about the pandemic can play a pivotal role in the adoption of mitigation strategies. Policy-driven large-scale test deployments (such as the SHIELD program) and their data-based evaluations (such as our study) are perhaps the best instruments to raise public awareness. Policymakers can benefit from these results by accounting for population behavior in making decisions regarding enrollment strategies. As indicated, the decision to adopt a consent model is left to individual school districts; however, having access to the results is likely to provide an objective basis for the decision on enrollment approaches in the future, for any further waves of the COVID-19 pandemic or other similar pandemics. An issue worth mentioning is the tension between safety and privacy concerns. Policymakers can design the implementation of testing programs to address some of the privacy concerns by using technology and secure data management processes.

### Limitations of the study

One limitation of the analysis is that the data are entirely from one state, Illinois. Also, the purpose of our analysis is to test the population-level effect of two different consent models of testing and not individual-level behavior in response to the two models. While the aggregate differences are manifestations of collective individual behavior, we acknowledge that there are individual variations in testing behavior. Notwithstanding the limitations, the access to a unique proprietary dataset from a large-scale implementation of the TTS program allows us to perform robust evaluation of the effect of two different enrollment approaches of testing. Future research might benefit from a detailed comparison of the two enrollment approaches, specifically examining the financial implications of their implementation. This includes analyzing the costs associated with testing equipment and materials, the duration required for testing, and the labor needed. Also, we recognize in our study’s limitations the uncontrollable variables, such as differential testing propensities among asymptomatic individuals and varying likelihoods of underreporting symptoms, which are critical in non-experimental settings. Our use of an instrumental variable approach partially addresses these issues, enhancing the robustness of our findings.

## STAR★Methods

### Key resources table

**Table:** 

REAGENT or RESOURCE	SOURCE	IDENTIFIER
**Software and algorithms**		

R (Version 4.1.1)	https://www.r-project.org/	RRID: SCR_001905
RStudio	https://www.rstudio.com/	RRID: SCR_000432
Lme4 (package)	https://cran.r-project.org/web/packages/lme4/index.html	RRID: SCR_015654
lmerTest (package)	https://cran.r-project.org/web/packages/lmerTest/index.html	RRID: SCR_015656
ggplot2 (package)	https://cran.r-project.org/web/packages/ggplot2/index.html	RRID: SCR_014601
mediation (package)	https://cran.r-project.org/web/packages/mediation/vignettes/mediation.pdf	NA

### Resource availability

#### Lead contact

Further information and requests for resources should be directed to and will be fulfilled by the lead contact, Dr. Anton Ivanov (antoniva@illinois.edu).

#### Material availability

This study did not generate new unique reagents.

#### Data and code availability

• The data reported in this study cannot be deposited in a public repository because of restrictions associated with its sensitive and proprietary nature.
• This paper does not report original code.
• Any additional information required to reanalyze the data reported in this paper is available from the lead contact upon request.

### Method details

#### Data

We use data provided by the SHIELD Illinois program (https://www.uillinois.edu/shield), a screening testing infrastructure developed and deployed by the University of Illinois System in collaboration with the Illinois Department of Public Health (IDPH). It employs a saliva-based RT-PCR test, which has been approved by the CDC. Additionally, we collected data on school characteristics from the COVID-19 School Data Hub.^29^ Community positivity and vaccination data were provided to us by IDPH. The former represents the community (zip code) level positivity, averaged over four weeks of observations, and is computed as the number of new infections detected per unit test conducted at a zip code. The latter is the percentage of the population vaccinated (received two doses) at the beginning of a week in the zip code where a school is located. Location classification (urban-rural RUCA codes) was obtained from the U.S. Department of Agriculture^30^ as used in prior research.^31^ We categorized the RUCA codes into metropolitan and non-metropolitan by combining micropolitan, small-town, and rural categories into the non-metropolitan class. Finally, to control for the unobserved effect of population-level propensity to test and adopt other safety measures such as masking, social distancing, and vaccination, we included county-level data on voting percentages for democratic and republican parties. Extant literature has associated population-level political affiliation with the adoption of safety measures and vaccination hesitancy during the COVID-19 pandemic.^32^

We excluded all schools with incomplete records (approximately 20% of schools). Further, we excluded the first two weeks of the Fall 2021 semester (the weeks of August 29 and September 5, 2021) due to low levels of testing at the beginning. We also removed outlier observations for schools that tested its population more than once a week (two schools). Finally, we removed all schools where the number of tests was less than 10 on average. Of the 348 schools in the original dataset, our final dataset contains 259 schools; 115 schools followed the opt-out model and the remaining 144 schools followed the opt-in model. The total number of students from the 259 schools is 173,010, of which 60,980 students were enrolled in the TTS program for at least part of the observation time frame. The data spans the period between September 12 and November 18, 2021, and includes information on 296,102 tests. We received institutional review board (IRB) approval from the University of Illinois Urbana-Champaign (UIUC).

#### Analysis

To study the effects of the consent model on COVID-19 testing rate and test positivity rate, we use a combination of different methodological approaches. First, to assess the differences in outcome between opt-in and opt-out consent models, we use model-free analysis using a series of boxplots, bar charts, and their corresponding descriptive statistics (Section “effect of consent model”). Second, to account for the effect of other relevant factors that may be associated with response variables, we conduct a regression analysis that includes the primary explanatory variable of interest, namely, consent model, and the other relevant confounding variables (Section “controlling for the heterogeneous effects of covariates”). However, there exists the potential for endogeneity in the data since the choice of consent model may systematically vary on factors such as community positivity and vaccination rates, which, in turn, are likely to be associated with both testing rate and test positivity at schools. Furthermore, we do not observe other relevant factors such as mask-wearing and social distancing. Third, to account for endogeneity originating from non-random assignment of schools adopting different enrollment approaches and omitted variable bias, we employ *(i)* matching by means of the coarsened exact matching (CEM) method (Section “heterogeneous effects”), *(ii)* a Heckman selection model, and *(iii)* an instrumental variable approach using two-stage least squares (Section “endogeneity corrected regression models”). Finally, to formally establish the causal pathway from the TTS consent model to school positivity rates through the testing rates, we conduct a graphical analysis of the testing rate and test positivity across schools, extend the matching analysis to account for the mediation effect of testing rate on test positivity, and estimate a formal model-based mediation analysis using a Bayesian mediation analysis (Section “explaining lower positivity rates via higher testing rates”).

### Quantification and statistical analysis

Statistical analyses were performed using R (Version 4.1.1, R Foundation, Vienna, Austria). The number of observation units (schools) is 259, and number of time-periods of observation is 10. All the statistical details are provided in the respective sections, figures and regression tables. The sample selection has been described in the STAR methods section of the paper. Significance was defined as follows: *0 < ‘∗∗∗’ <= 0.001 < ‘∗∗’ <= 0.01 < ‘∗’ <= 0.05 < ‘+’ <= 0.1.*
